# Nutritionally Enriched Maize- and Rice-Based Gluten-Free Biscuits: Leveraging Local Legume Flours for Improved Quality

**DOI:** 10.3390/foods14173050

**Published:** 2025-08-29

**Authors:** Wafa Allouch Tounsi, Hajer Debbabi, Nesrine Hadj Yahia, Youkabed Ouederni Zarroug, Haifa Sebii, Leila Doggui, Mariem Bouhadida, Ali Ouji, Mohamed Kharrat, Dorra Sfayhi Terras

**Affiliations:** 1 Field Crop Laboratory (LR16INRAT02), National Institute of Agronomic Research of Tunisia, University of Carthage, Rue Hedi Karray, Ariana 2049, Tunisia; allouchwaf@gmail.com (W.A.T.);; 2 Food Science and Technology Department (UR17AGR01), National Agronomic Institute of Tunisia, University of Carthage, 43 Charles Nicolle Avenue, Tunis 1082, Tunisia; hajer.debbabi@inat.ucar.tn; 3 Laboratory of Analysis, Valorization, and Food Safety, University of Sfax, ENIS, Sfax 3038, Tunisia; 4 Competitiveness pole of Bizerte, Union du Grand Maghreb Arabe boulevard, Bizerte 7080, Tunisia

**Keywords:** legume flour, biscuit, gluten-free, mixture design, sensory evaluation, nutritional profile, total polyphenols

## Abstract

Gluten-free (GF) baked goods often lack nutritional balance due to the limited protein and fiber content of standard cereal flours like rice and maize. A mixture design methodology was used to evaluate the interaction effects between cereal and legume flours on the physical and textural properties of the biscuits, including hardness, water activity (aw), CIE color parameters (L*, a*, and b*), spread ratio, and baking loss. The results indicated that incorporating legume flour, particularly chickpea flour, significantly increased biscuit hardness (from 22.00 N to 34.66 N) and reduced water activity (from 0.23 to 0.17). All three legume flours reduced the spread ratio, with chickpea flour having the most pronounced effect (from 4.91 to 4.75). Nutritionally, the inclusion of legume flours improved the protein (from 6.46 g/100 g to 11.90 g/100 g), mineral (from 0.58 g/100 g to 1.25 g/100 g), fiber (from 15.73 g/100 g to 21.13 g/100 g), and polyphenol contents (0.34 mg GAE/g compared to 0.18 mg GAE/g for the control). Moreover, DPPH scavenging activity was significantly higher (72.72% vs. 31.49% for the control). Sensory evaluations indicated that the inclusion of legume flours positively affected the biscuits’ overall sensory attributes, especially appearance, but had a minor negative effect on texture. This study aimed at utilizing local legume flours: faba bean, chickpea, and lentil, besides the traditional standard flours: rice and maize, to develop nutritious and flavorful gluten-free biscuits. These results highlighted the use of combinations of local legume with cereal flours to produce GF biscuits with improved physical, sensory, and nutritional qualities.

## 1. Introduction

Biscuits are favored bakery items due to their convenience, reasonable cost, and commendable nutritional profile. The rising prevalence of wheat flour protein allergies, such as celiac disease, has prompted the development of diverse gluten-free (GF) products. The ingestion of gluten peptides in celiac disease patients provokes an abnormal immune reaction, resulting in distinctive intestinal tissue damage marked by villous atrophy, hyperplasia of the crypts, and increased populations of lymphocytes within both the intraepithelial layer and the lamina propria [1]. Adherence to a GF diet typically results in the restoration of normal mucosal histology and the remission of clinical symptoms. However, maintaining strict compliance with this dietary regimen is challenging, and patients frequently experience increased health concerns and reduced nutritional value in their food intake [2,3].

Usually, the standard flour and starch extensively used as a substitute for wheat flour in the formulation of GF bakery products are maize and rice. Nonetheless, these flours and starches are typically not enriched or fortified, and the resulting GF products follow suit [4]. In addressing this issue, various nutrient-dense alternative raw materials have been employed to enhance nutritional quality and broaden the formulations of GF products. Legume flour, being naturally GF, holds potential for various uses in boosting the nutritional content of GF food items like pasta, breads, and snacks [5]. Healthy and sustainable diets are becoming more important, and using local resources is a key part of this. By adding local legumes like chickpea, faba bean, and lentil flours to GF biscuits, we can improve nutrition. This reduces the need to import ingredients and helps create a more sustainable food system. It also supports local economies, agriculture, and biodiversity. Furthermore, legume flours offer many essential amino acids, including lysine, leucine, isoleucine, and phenylalanine. When pulses like peas and beans are mixed with grains such as wheat and rice, they provide a balanced essential amino acid profile [6]. Chickpea (*Cicer arietinum* L.) is among the earliest and most extensively consumed legumes globally. Chickpea and other pulse grains play a role in ensuring food security, nutrition, and health by serving as valuable sources of essential nutrients like protein, dietary fiber, minerals, and bioactive compounds. Besides its nutritional benefits, chickpea flour possesses interesting functional properties, including emulsifying and foaming characteristics, as well as a high water and oil absorption capacity, making it a promising ingredient for a variety of food products [7]. In addition, it is acknowledged as a cost-effective and wholesome vegetarian food option, thanks to its advantageous medicinal properties. Studies of GF biscuits using chickpea flour in the formulations have shown promising results [8]. Other researchers have reported good results when producing GF biscuits employing a flour basis of chickpea [9,10,11] in the proportion of 12.5%, 20% and 40%, respectively, in combination with rice flour. Furthermore, legumes like bean and lentil flour have been used as an alternative to substitute gluten-containing flour in the formulation of GF bakery products due to their advantageous nutritional composition for human consumption, being low in fat and rich in proteins, dietary fibers, iron, zinc, and vitamins [12]. Additionally, their amino acid profile can complement the attributes of cereal flours, thereby enhancing the protein biological value of the flour blend [13]. The incorporation of faba bean in the formulation of biscuits has already been studied [14,15,16], resulting in a successful food product.

In recent years, there has been a growing trend in the consumption of lentils. This can be attributed to an improved understanding of their excellent nutritional composition and potential health benefits, including a reduced risk of chronic diseases such as obesity, type-2 diabetes, hypertension, cancer, and cardiovascular diseases. This is particularly evident in the elderly population of the Mediterranean region [17]. A significant application of lentils in the food industry involves their use in the form of flour. This flour is widely utilized as a thickener, binder, gelling agent, and/or stabilizer in a diverse array of food products, thanks to its functional properties [18]. Despite their advantageous properties, lentils are still relatively underutilized in GF biscuit products. Recent studies have investigated the use of lentil flour in cookie formulations, demonstrating enhancements in both textural and physical properties [19,20]. Therefore, lentil flours can be promising raw materials for GF bakery products depending on physicochemical and sensory characteristics.

The present study aimed to explore the synergistic effects of incorporating locally sourced and readily available legume flours, specifically faba bean, lentil, and chickpea, in combination with traditional GF cereal flours (rice and maize) for the development of innovative GF biscuits.

## 2. Materials and Methods

### 2.1. Plant Materials

The GF flours selected in our study were faba bean, chickpea, rice, lentil, and maize. All the raw materials, faba beans (variety “Bachar”), chickpeas (variety “Beja1”), and lentils (variety “Kef”), were supplied by the agricultural experimentation unit of the National Institute of Agronomic Research of Tunisia (INRAT). Faba beans and lentils were hulled and then roasted with chickpeas in a convection oven (De Dietrich, Reichshoffen, Alsace, France) at 200 °C for 7 min (lightly modified from [21]). Subsequently, all the legume seeds were finely ground into flour (using a 0.5 mm sieve) employing a grinder (Retschmuhle, 7311 Dettigen-Tech, West Germany). Maize seeds purchased from a local market were sorted and ground using the same grinder. Similarly, for rice, seeds purchased from a local market were ground using a laboratory grinder (Cyclotec, Foss). Following this, the flours were packed into glass containers and stored in a fridge (4 °C) for further use. Other baking ingredients included egg, fat, sugar, vanilla sugar, baking powder, sodium bicarbonate, and salt, which were purchased locally.

### 2.2. Raw Material Characterization

The flour samples were analyzed to determine their physicochemical properties. The moisture, ash, fat, and protein (total nitrogen N × 6.25 for legumes and N × 5.7 for cereals) contents were ascertained using standard AOAC methods [20]. The carbohydrate content was calculated by difference (100%—[protein% + ash% + moisture% + fat %]).

The total polyphenol content (TPP) was measured using the Folin–Ciocalteu colorimetric method [22]. To prepare the sample, polyphenols were first extracted from the material using methanol as solvent. The absorbance was measured at 760 nm against distilled water as a blank. Gallic Acid (GA) was used as a standard reference to generate a calibration curve; the TPP was quantified and expressed as mg of GA equivalents per g of dry matter of the sample (mg GAE/g DM sample) through the calibration curve of GA.

The levels of iron (Fe), zinc (Zn), and potassium (K) were determined through inductively coupled plasma optical emission spectrometry (ICP-AES) using an atomic emission spectrometer (Perkin Elmer/Avio 200, USA). Prior to the ICP-AES measurement, the samples underwent mineralization by treatment with HNO_3_ and H_2_O_2_ at 100 °C until complete digestion was achieved. These procedures adhered to the standard methods outlined in LST EN 1551 [23].

Color measurements of the flour samples were performed in triplicate using a Chroma meter CR-400 (Minolta, Japan), recording the CIE L*, a*, and b* values. The L* value represents lightness on a scale from 0 to 100, indicating darkness to lightness. The a* value indicates the red–green color spectrum, where a higher positive a* value suggests more red. The b* value represents the yellow–blue color spectrum, with a higher positive b* value indicating more yellow.

### 2.3. Preparation of Biscuits

The GF biscuit formulation consisted of varying proportions of the five GF flours (faba bean, lentil, chickpea, rice, and maize), whole fresh egg at room temperature, margarine (Jadida brand), sugar, sugar vanilla (Vanoise brand), sodium bicarbonate, baking powder (Vanoise brand, composed of sodium acid carbonate, disodium diphosphate (leavening agents), and starch), and salt. The control biscuit formulation was composed solely of rice flour (70 g/100 g) and maize flour (30 g/100 g). This formula was developed according to the method outlined by Tyagi et al. [24] with slight adjustments. The GF biscuits were prepared following a standardized procedure. Initially, a homogeneous and creamy batter was achieved through the creaming method, wherein egg, fat, sugar, vanilla sugar, and salt were combined using a stand mixer (Kenwood KHC29, Kenwood Limited, Havant, Hampshire, UK) equipped with a K-beater attachment and mixed for 2 min at a medium speed. Subsequently, the dry ingredients, comprising baking powder, sodium bicarbonate, and the specified flour blend, were gradually incorporated into the wet mixture and blended for an additional 6 min until a uniform dough was formed. The resulting dough was then rested in a refrigerator at 4 °C for 20 min. Following the resting period, the dough was sheeted to a consistent thickness of 5 mm using a rolling pin and cut into circular shapes with a 40 mm diameter mold. The biscuits were baked in a preheated convection oven (De Dietrich, Reichshoffen, Alsace, France) at 175 °C for 20 min, subsequently cooled at room temperature for 30 min, and kept in hermetic containers to maintain their integrity.

### 2.4. Optimization of the GF Biscuit Recipe

The extreme vertices mixture design was used to optimize the proportions of the five GF flours added in the recipe of GF biscuits: faba bean (X1), lentil (X2), chickpea (X3), rice (X4), and maize (X5). The proportions of components were represented as fractions of the mixture, with a total sum (X1 + X2 + X3 + X4 + X5) equal to 100. The D-Optimal Mixture Design was implemented to explore the interactions among faba bean, lentil, chickpea, rice, and maize flours, and their impact on the technological and nutritional properties of the enriched products. The experimental range was confined to 10–25% for faba bean, 5–20% for lentil, 5–20% for chickpea, 24.5–56% for rice, and 10.5–24% for maize. These limits were established because deviating from this range resulted in a bitter aftertaste after baking and the distinctive beany flavor of legume flour. The study’s experimental design involved formulating 23 distinct biscuit combinations, each presenting diverse levels of faba bean, lentil, chickpea, rice, and maize flour.

### 2.5. GF Biscuits Quality Characterization

#### 2.5.1. Physical Characterization

The diameter and thickness of the biscuits were determined using a caliper (ACEM Outillage, AC304B1. W-1220, Sfax, Tunisia) on a set of 6 biscuits. Spread ratio was calculated by dividing the diameter by the thickness. Weight was determined using an electronic precision balance (Mettler Toledo, Switzerland). The baking loss (%) was determined using Equation (1).

(1) $$Baking\;loss\left(\%\right)=\frac{\left(Wf-W0\right)}{W0}\times 100$$

Wf: Biscuit weight after baking.

W0: Biscuit weight before baking.

Biscuit texture was carried out by Texture Profile Analysis (TPA) using a three-point bending test with the HDP/3PB rig and a speed of 1 mm/s, at room temperature using a texture analyzer TAXT2i (TVT 6700, Perten Instruments, Warrington, UK). All analyses were performed in triplicate for each biscuit formulation. Through this analysis, we were able to determine the hardness and fracturability of the biscuits. Dough hardness, cohesiveness, springiness (mm), and adhesiveness (N) were established through a Texture Profile Analysis (TPA) test conducted at 25 °C utilizing a texture analyzer (LLOYD instruments, Fareham, UK). Compression of the samples was carried out using a cylindrical probe (20 mm in diameter) at 50% deformation, with a displacement speed of 30 mm/min.

Water activity (aw) was measured at 25 °C employing an apparatus (Novasina Aw Sprint TH-500, Axair Ltd., Pfäffikon, Switzerland). Color measurements of the biscuits were conducted three times as described previously using the Chroma meter CR-400 (Minolta, Japan) based on the CIE L*, a*, and b* values.

#### 2.5.2. Nutritional Characterization

Proximate composition of the GF biscuits (moisture, ash, proteins, and fat) was assessed using the standard AOAC methods [25] following the same procedure as for the raw flours. Total dietary fiber (TDF) and insoluble dietary fiber (IDF), reported as g TDF or IDF per 100 g of dry matter, were determined using the enzymatic–gravimetric method [25]. Carbohydrates were calculated by difference. The TPP was determined using a Folin–Ciocalteu assay, following the same procedure as for the raw flours.

The DPPH radical scavenging activity was determined as described by Kedare et al. [26] and Marteau et al. [27]. The experiment utilized a DPPH concentration of 0.1 mM (4 mg of the free radical in 100 mL of methanol). A control sample of DPPH solution was prepared by mixing 2.0 mL of the solution and 1.0 mL of methanol. The samples were prepared by mixing 2.0 mL of DPPH solution and 1.0 mL of the extracts previously prepared for the PTT analysis. The mixture was left at ambient temperature for 30 min in the dark. Scavenging activity was measured spectrophotometrically by monitoring the decrease in absorbance at 517 nm. DPPH radical scavenging activity was calculated using Equation (2):

(2) $$DPPH\;radical\;scavenging\;activity\left(\%\right)=\frac{Acontrol-Aextract}{Acontrol}\times 100$$

where Acontrol is the absorbance of the control (without the sample extract), and Aextract is the absorbance of the sample extract.

### 2.6. Sensory Evaluation

The sensory attributes of the optimized GF biscuit formulation and the standard GF biscuit were evaluated by a panel of 60 untrained participants (22 men, 38 women; age range 18–60 years). Informed consent was obtained from all subjects involved in the study. The sensory evaluation was conducted in a controlled environment. Each biscuit sample was coded with a unique three-digit random number and presented to the panelists in a randomized order to minimize potential bias due to presentation order or sample carry-over effects. The panelists were directed to assess the biscuits based on the following sensory attributes: appearance, color, odor, texture, taste, crispiness, aftertaste, and overall acceptability. A 7-point hedonic scale was employed for the evaluation of each attribute, ranging from 1 (dislike extremely) to 7 (like extremely). To minimize residual sensory effects between sample evaluations, the panelists were provided with water as a palate cleanser.

### 2.7. Statistical and Data Analysis

Experimental design and analysis were carried out using Minitab19^®^ Statistical Software (Minitab Ltd., Coventry, UK). In this study, the linear (Equation (3)), quadratic (Equation (4)), special cubic (Equation (5)), and full cubic models (Equation (6)) were fitted to the experimental data. The selection of the appropriate model for each response was based on the fit quality using criteria such as the coefficient of determination (R^2^), the adjusted coefficient of determination (R^2^_Adj_), and the significance level of regression (*p* < 0.05).

(3) Y=β1X1+β2X2+β3X3+β4X4+β5X5

(4) $$\begin{array}{l} Y=\beta 1X1+\beta 2X2+\beta 3X3+\beta 4X4+\beta 5X5+\beta 12X1X2+\beta 13X1X3+\beta 14X1X4\\ \;\;\;\;\;\;+\beta 15X1X5+\beta 23X2X3+\beta 24X2X4+\beta 25X2X5+\beta 34X3X4\\ \;\;\;\;\;\;+\beta 35X3X5+\beta 45X4X5 \end{array}$$

(5) $$\begin{array}{l} Y=\beta 1X1+\beta 2X2+\beta 3X3+\beta 4X4+\beta 5X5+\beta 12X1X2+\beta 13X1X3\\ \;\;\;\;\;+\beta 14X1X4+\beta 15X1X5+\beta 23X2X3+\beta 24X2X4+\beta 25X2X5\\ \;\;\;\;\;+\beta 34X3X4+\beta 35X3X5+\beta 45X4X5+\beta 123X1X2X3\\ \;\;\;\;\;+\beta 124X1X2X4+\beta 125X1X2X5+\beta 234X2X3X4\\ \;\;\;\;\;+\beta 235X2X3X5+\beta 345X3X4X5 \end{array}$$

(6) $$\begin{array}{l} Y=\beta 1X1+\beta 2X2+\beta 3X3+\beta 4X4+\beta 5X5+\delta 12X1X2(X1-X2)\\ \;\;\;\;\;\;\;+\delta 13X1X3(X1-X3)+\delta 14X1X4(X1-X4)+\delta 15x1X5(X1\\ \;\;\;\;\;\;\;-X5)+\delta 23X2X3(X2-X3)+\delta 24X2X4(X2-X4)\\ \;\;\;\;\;\;\;+\delta 25X2X5(X2-X5)+\delta 34X3X4(X3-X4)+\delta 35X3X5(X3\\ \;\;\;\;\;\;\;-X5)+\delta 45X4X5(X4-X5)+\beta 123X1X2X3+\beta 124X1X2X4\\ \;\;\;\;\;\;\;+\beta 125X1X2X5+\beta 234X2X3X4+\beta 235X2X3X5\\ \;\;\;\;\;\;\;+\beta 345X3X4X5 \end{array}$$

where Y is the predictive dependent variable (biscuit hardness, spread ratio, baking loss, aw, L*, a*, and b*). The component proportions X1, X2, X3, X4, and X5 were expressed as fractions of the mixture with a sum of 100; β1, β2, β3, β4, and β5 are coefficients associated with the factors faba bean, lentil, chickpea, rice, and maize percentage, respectively. Additionally, β12, β13, β14, β15, β23, β24, β25, β34, β35, β45, β123, β234, β235, β345, δ12, δ13, δ14, δ15, δ23, δ24, δ25, δ34, δ35, and δ45 are the equation coefficients linked to the interactions among the five factors. Coefficients exhibiting positive values signify synergistic effects, while negative values indicate antagonistic effects between the ingredients [28]. Utilizing the regression model significance, contour plots were then generated to identify optimal blend regions. The finest formulations were subsequently selected to reduce the hardness of GF biscuits to a minimum while maintaining the target values for the other responses, aiming for products that meet sensory acceptance criteria. The data were presented as mean ± standard deviations based on three samples. To compare the physical properties and proximate composition of GF flours and biscuits, an analysis of variance (ANOVA) was conducted using SPSS (IBM SPSS Statistics Version 25) following the one-way ANOVA and Tukey’s test at the level of *p* < 0.05.

## 3. Results and Discussion

### 3.1. Flour Characterization

The proximate composition and color parameters of the studied GF flours are presented in Table 1. Significant differences (*p* < 0.05) in the macronutrient profiles were observed between the legume flours (faba bean, chickpea, and lentil) and the cereal flours (rice and maize).

The moisture content ranged from 10.40% (chickpea flour) to 13.36% (rice flour). Interestingly, the legume flours had significantly higher protein (22.67% to 28.71%) and ash contents (2.37% to 2.64%) compared to the cereal flours (10.26% to 10.42% protein, 0.71% to 0.95% ash). These results align with previous findings on pulse flours, highlighting their superior nutritional profile compared to commonly used cereal flours in GF formulations [29,30]. These higher protein contents in the legume flours (+27%) can enhance the nutritional value of GF products, which are often criticized for their lower protein levels compared to their wheat-based counterparts.

Conversely, the carbohydrate content was notably higher in the cereal flours (71.90% to 75.25%) compared to the legume flours (55.72% to 59.22%). In terms of fat content, the chickpea flour had the highest value (5.06%), while the rice flour showed the lowest (0.4%). Low fat values were found in legume flours: respectively, 1.6% and 1.88% for the lentil and faba bean flours. These results were in agreement with the findings of Millar et al. [30] on the low fat content of pulse flours (below 2%), including those from faba bean, green pea, and yellow pea.

According to Table 1, TPP was highest in the maize flour (1.04 mg GAE/g DM), followed by chickpea (0.65 mg GAE/g) and faba bean (0.43 mg GAE/g), and lowest in the rice flour (0.2 mg GAE/g DM). Our findings are consistent with the studies by Di Cairano et al. [31] and Millar et al. [29], highlighting elevated values in legume flours, contributing to their potential antioxidant properties.

Mineral analyses revealed that the lentil flour had the highest iron content (152.45 ppm), the chickpea flour was richest in zinc (51.28 ppm), and the faba bean flour had the highest potassium concentration (1.10%). Similar results were found in pulse flours by De Angelis et al. [32] and Millar et al. [30], suggesting that incorporating legume flours can effectively address potential micronutrient deficiencies often associated with strict GF diets based primarily on refined cereal flours.

The CIE color analysis (L*, a*, and b*) showed significant variation in the L* values amongst the flour samples (Table 1). Chickpea had the highest value (93.81) with no significant difference between the rice and faba bean flours (*p* < 0.05), implying the brightest color. This provides a strong reason to use such flour as a substitute for standard ones without altering the color of the final product. In contrast, maize had the lowest L* (85) with no significant difference from lentils. The chickpea flour also had the highest negative a* value (–2.57), indicating a more greenish hue, while the maize flour exhibited the most yellowish tint (b* = 39.39). These findings suggest that the chickpea and lentil flours may enhance the visual appeal of GF biscuits without significantly altering their color, and thus, compromising consumer acceptance.

Altogether, the characterization of the raw flours has clearly demonstrated the nutritional advantages of incorporating legume flours into GF formulations. Their significantly higher protein, ash, and in some cases, polyphenol contents, place them as valuable ingredients for enhancing the nutritional profile of GF products that traditionally rely on less nutrient-dense cereal flours such as rice and maize.

### 3.2. Optimization of Gluten-Free Biscuits Enriched with Legume Flour

#### 3.2.1. Experimental Design Methodology

To assess the impact of varying inclusion rates of the faba bean, lentil, chickpea, rice, and maize flours on diverse responses such as GF biscuit hardness, spread ratio, baking loss, aw, L*, a*, and b*, a D-optimal mixture design was employed to optimize the 5-factor design. Table 2 presents the 23 test results conducted based on the factorial model that specifies the combinations of different factor levels. The “backward elimination regression” method was employed, involving the removal of non-significant interactions. Only variables evaluated as significant at *p* < 0.05 level were chosen for constructing the predicted models. Other parameters were evaluated, including fracturability (mm), dough cohesiveness, springiness (mm), and adhesiveness (N). However, they were excluded from building the predicted models as they were deemed statistically insignificant (*p* > 0.5). Among the responses, the linear model best fits biscuit hardness, while the full cubic model was optimal for color parameters (L*, a*, and b*). The special cubic model was the best fit for baking loss and aw. Equations (7)–(13) depict the predicted model for each dependent variable.

(7) Biscuit hardness = 0.96X1 + 0.79X2 + 1.22X3 + 0.3X4 + 0.22X5

(8) Spread ratio = −0.46X1 − 0.13X2 − 6.98X3 + 0.3X4 + 4.67X5 + 0.11X1X2 − 0.18X1X3 − 0.12X1X4 + 0.13X1X5 − 0.17X2X3 − 0.12X2X4 + 0.12X2X5 − 0.04X3X4 + 0.21X3X5 + 0.25X4X5 + 0.006X1X2(X1 − X2) + 0.002X1X3(X1 − X3 − 0.01X1X5(X1 − X5) − 0.0001X3X4(X3 − X4)

(9) Baking loss = 25.11X1 + 29.31X2 − 23.19X3 + 2.78X4 + 25.57X5 − 1.81X1X3 − 0.05X1X5 + 0.08X2X3 + 0.006X2X4 − 0.05X2X5 + 0.08X3X4 + 0.01X3X5 − 0.06X4X5 + 0.001X1X2(X1 − X2) − 0.003X1X3(X1 − X3) + 0.002X1X5(X1 − X5) + 0.0001X2X4(X2 − X4) + 0.0002X3X4(X3 − X4)

(10) aw = − 0.41X1 − 0.36X2 − 0.03X3 − 0.01X4 − 0.01X5 + 0.02X1X2 + 0.006X1X3 + 0.006X1X4 + 0.006X1X5+ 0.006X2X3 + 0.005X2X4 − 0.0003X1X2X3 − 0.0003X1X2X4 − 0.0002X1X2X5 + 0.00001X1X3X5

(11) L* = − 9.1X1 − 0.02X2 + 6.29X3 + 2.89X4 − 5.02X5 + 0.11X1X2 + 0.12X1X4 + 0.21X1X5 − 0.07X2X3 + 0.08X2X5 − 0.06X3X4 + 0.03X4X5 + 0.001X1X2(X1 − X2) + 0.004X1X3(X1 − X3) + 0.001X1X4(X1 − X4) − 0.002X1X5(X1 − X5) + 0.001X2X4(X2 − X4) + 0.001X3X4(X3 − X4)

(12) a* = 3.87X1 − 6.99X2 − 2.69X3 + 0.01X4 + 2.93X5 +0.06X1X2 − 0.06X1X4 − 0.09X1X5 + 0.11X2X3 + 0.08X2X4 + 0.04X2X5 + 0.02X3X4 − 0.03X4X5 − 0.003X1X2(X1 − X2) − 0.002X1X3(X1 − X3) − 0.0001X1X4(X1 − X4) + 0.002X1X5(X1 − X5) + 0.0001X2X4(X2 − X4) − 0.0001X3X4(X3 − X4)

(13) b* = 1.28X1 − 6.07X2 − 0.07X3 + 0.22X4 + 1.97X5 + 0.09X1X2 − 0.03X1X4 − 0.02X1X5 + 0.06X2X3 + 0.07X2X4 + 0.07X2X5 − 0.004X1X2(X1 − X2) − 0.002X1X3(X1 **−** X3) − 0.0002X1X4(X1 − X4) + 0.006X1X5(X1 − X5) + 0.0002X2X4(X2 − X4)

The validation of this model was based on the verification of the linear Pearson coefficient of determination of the model (R^2^) and the ANOVA represented in Table 3. In statistics, the linear Pearson coefficient of determination, denoted as R^2^, serves as an indicator of the accuracy in predicting linear regression. A lower R^2^ (close to 0) signifies greater dispersion of data points around the regression line, while a higher R^2^ (tends toward 1) indicates a tighter clustering of points around the regression line [33]. All the models exhibited satisfactory coefficients of determination (R^2^) and adjusted (R^2^_Adj_) values presented in Table 3.

#### 3.2.2. Effect of the Different Flours on the Studied Responses

The results from the current investigation indicated that the five factors (faba bean, lentil, chickpea, rice, and maize flours) have a notably significant impact on biscuit hardness, spread ratio, baking loss, aw, L*, a*, and b*.

**Biscuit hardness**

**Figure 1:**
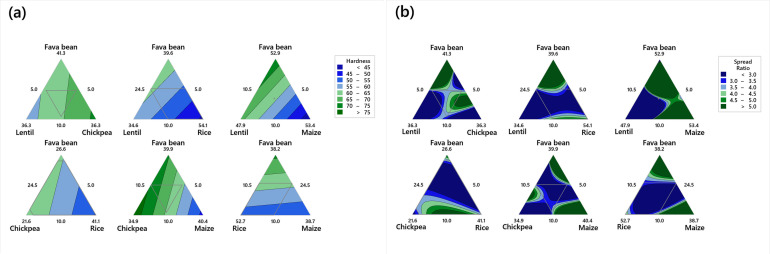

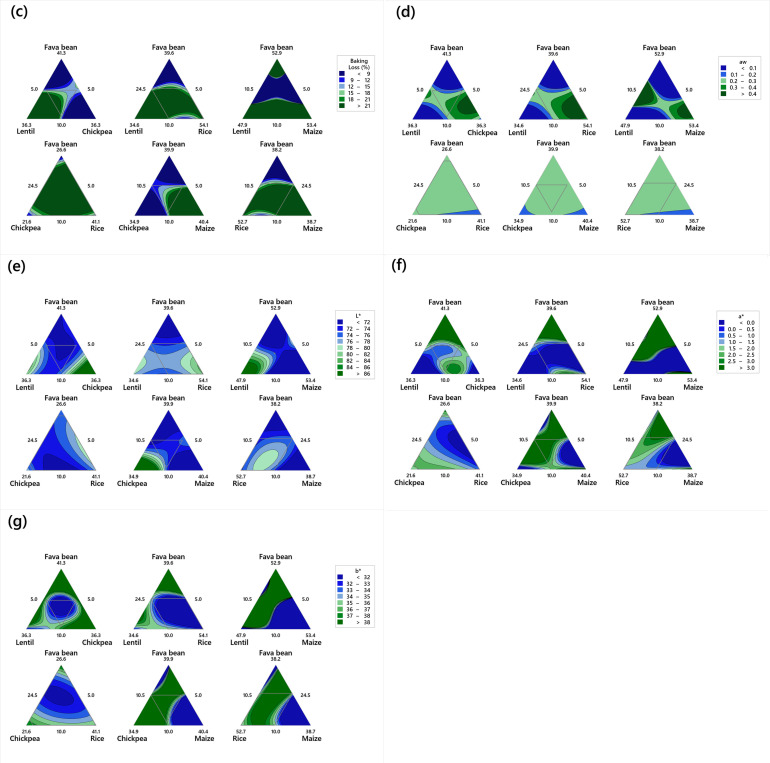
Contour plots for predicted (**a**) biscuit hardness (N); (**b**) spread ratio; (**c**) baking loss (%); (**d**) aw; (**e**) L*; (**f**) a*; (**g**) b*.

Hardness is a critical textural property, reflecting the resistance of biscuits to deformation. Specifically, in bakery items, it exhibits a robust correlation with consumers’ perception of product freshness [34]. As shown in Table 2 and in Figure 1a, the incorporation of the legume flour significantly increased biscuit hardness (*p* < 0.05), with the chickpea flour having the most pronounced effect. Among all the single ingredients evaluated, the chickpea flour exhibited the highest coefficient on the regression models (Equation (7)), indicating the greatest contribution to increased hardness, whereas maize, which had the lowest coefficient, was associated with reduced hardness. As illustrated in the contour plot (Figure 1a), the dark green region indicates that chickpea exerts the most pronounced positive effect on cookie hardness, with values exceeding 70 N, followed by faba bean and lentil. Similarly, the remaining flours exhibited a significant positive (*p* < 0.05) effect on hardness, with the rice flour having the least coefficient (Equation (7)). This increase is attributed to the high protein and fiber content of the legume flour, which reduces starch hydration and gelatinization, forming a denser structure [35]. Additionally, the proteins in chickpea exhibit a strong gelling capacity [36,37], creating a robust gel structure that could potentially impact and elucidate the rise in hardness in GF biscuit formulations based on the chickpea flour. These results are in line with those found by Santos et al. [38], who studied the effect of adding chickpea flour on the crumb hardness of bread.

**Spread ratio**

The spread ratio plays a crucial role in assessing the appearance quality of cookies [39]. The different GF biscuit formulations showed a spread ratio ranging from 3.09 to 4.26 (Table 2). The spread ratio decreased with the inclusion of the legume flour. The chickpea flour exhibited the strongest negative effect. In contrast, rice and maize had a significant positive effect, especially the maize flour, which contributes to the increase in the spread ratio (Equation (8)), maintaining the desired biscuit dimensions. Those results were confirmed by the contour plots (Figure 1b) and could be explained by the competition among the legume/cereal flour for available water for dough consistency. The inclusion of the legume flour may increase the number of hydrophilic sites available to compete for the limited free water in biscuit dough [40], which could contribute to a reduction in the spread ratio. The results of this study are consistent with previous findings [41,42,43], indicating a reduction in the spread ratio for cookies or biscuits with the inclusion of chickpea and pigeon pea flours as substitutes. Similarly, Yadav et al. [44] found that the spread ratio decreased with the addition of plantain and chickpea flour in the formulation of biscuits.

**Baking loss**

Baking loss, an indicator of moisture and gas release during baking, was significantly influenced by flour composition. This loss may be attributed to the escape of gas during the baking process. The chickpea flour reduced baking loss, while the faba bean, lentil, maize, and rice flours increased it (Equation (9)). The binary and ternary flour combinations varied between synergic and antagonist effects. Those results were confirmed by the contour plots (Figure 1c). This could be explained by the new structural network formed with the legume flour. The ability of legume flours to retain moisture is attributed to their higher water-binding capacity, as confirmed by prior research [45]. This capacity is determined by the starch and protein content, along with the flour’s structure and composition. Factors such as starch content, the amylose-to-amylopectin ratio, and the size and structure of starch granules can influence water binding. Proteins that create an elastic network during dough preparation improve the dough’s ability to retain gas, which helps prevent volume loss during baking [20]. The results suggest that legume flours have a similar water-binding capacity compared to rice and maize flours, but they exhibit a lower ability to form elastic dough. In this line, Ammar, Gharsallah [46] noted that the baking loss decreased by the presence of whey protein concentrate in the formulation of GF sponge cake, and the effect was more pronounced at 15% especially.

**Water activity aw**

Analyzing the aw of food products is highly valuable, not just for predicting their shelf life but also for gaining insights into their textural qualities [47]. Water activity decreased significantly with the incorporation of the legume flour, particularly faba beans and lentils (Equation (10)). This decrease might be attributed to the water-binding capacity of proteins. The use of composite flours leads to the formation of aggregates with an increased density of hydrophilic sites, which enhances competition for the limited free water present in the dough [48,49]. Additionally, proteins tend to undergo gelation, which facilitates the entrapment of water within the gel matrix, thereby further reducing aw that could play an important role in the extended storage life of biscuits by reducing microbial spoilage risks [50]. All the binary combinations had a significant positive effect on aw, while ternary combinations had an antagonist effect on aw by decreasing its values. The contour plots for predicted aw confirmed those results (Figure 1d). In this line, Ammar, Gharsallah [46] noticed a decrease in water activity of GF cakes prepared with rice and maize enhanced with whey protein concentrate incorporation. A comparable decrease in water activity was observed in soy protein-enriched cookies as the level of protein supplementation increased [49].

**Color parameters**

Evaluation of biscuit color is very important to determine their acceptability by the consumer. The lightness L* of biscuits increased by the incorporation of the rice and chickpea flour, while faba bean, maize, and lentil decreased L*. The results found in Table 1 confirmed those findings, where the rice and chickpea flours presented the highest L* (Equation (11)). Likewise, Bouasla et al. [51] displayed that the lightness of dry pasta samples decreased with an increase in the amount of lentil flour in the recipe. The faba bean, maize, and rice flours increased the redness of the GF biscuits, showing positive coefficients (Equation (12)), while chickpea and lentil presented a significant negative effect on a*. For yellowness, maize followed by faba bean flour had significant positive effects on this response, whereas lentil decreased b* (Equation (13)) by its negative coefficient, followed by chickpea flour. The contour plots for predicted L*, a*, and b* confirmed those results (Figure 1e–g). Similarly, Jayasena et al. [52] reported a constant increase in the b* values for dough and biscuits, with an increase in the lupine flour concentration in the formulation. These results suggest that the choice of legume flour can be tailored to achieve specific color attributes in GF biscuits.

**Desirability study**

The processing of D-Optimal Mixture Design using the Minitab software facilitated the simultaneous optimization of seven responses. This entirely numerical procedure enables the mathematical exploration of the combined effects of formulation parameters, aiming for the best possible values for the desired responses. Target values for each parameter are given in Table 3. The desirability (D) is at its minimum when the provided response is unsuitable and reaches its maximum when the given answer is highly satisfactory. For biscuit textural properties, the desirability was programmed to be at 100% by decreasing biscuit hardness to its minimum, with a desirability of 63.35% (Table 4). The other parameters were optimized, trying to obtain similar properties to the reference. Table 4 presents the component values of the GF biscuit formulation, achieving desirability (D) of 73.01%. The optimization of the different flour proportions was conducted to formulate an acceptable GF biscuit with satisfactory culinary quality and desirable texture properties. Based on the analysis of Table 4, the optimal formula consists of 24.50% of faba bean flour, 19.92% of lentil flour, 6.89% of chickpea flour, 24.67% of rice flour, and 24% of maize.

### 3.3. Physicochemical Analysis of the GF Optimum Biscuit Compared to the Control

Table 5 displays the physicochemical composition of the GF biscuits in comparison with the controls composed of 100% rice flour and maize. Our findings clearly demonstrated that the incorporation of legume flour in the optimized formulation resulted in a significant enhancement of the nutritional profile compared to the cereal-based control. Interestingly, the protein, fiber, ash, and total polyphenol contents were higher in the optimized sample, while the carbohydrate levels were reduced compared to the control. This improvement in nutritional composition can be directly attributed to the inherent nutritional characteristics of the incorporated legume flours (as shown in Table 1), which are notably richer in these components compared to the GF cereal flours, such as rice and maize, predominantly used as standard GF flours in the production of GF bakery products. Those results were in line with those of Yadav et al. [44], who reported a significant increase in ash, protein, and fiber in biscuits supplemented with chickpea and plantain. Our results clearly showed a great increase in the antioxidant activity (DPPH) of the optimized biscuit (72.72%) in comparison with the control (31.49%), highlighting the functional benefits derived from enrichment with legume flours, which are sources of bioactive compounds with antioxidant properties [29,31]. These results are consistent with the outcomes of the study by Thongram et al. [53] demonstrating a significant enhancement in the DPPH radical scavenging activity of cookies resulting from the partial substitution of wheat with legume flour, supporting the positive impact of legume incorporation on the antioxidant properties of cereal-based products.

**Figure 2:**
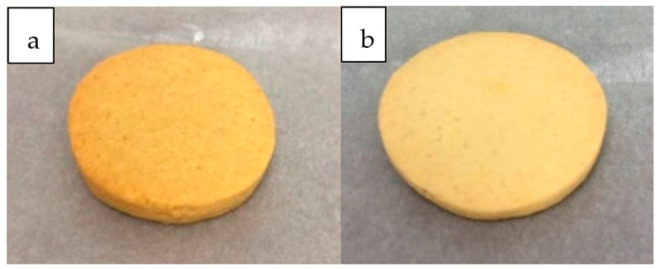
GF biscuits: (**a**) optimum and (**b**) control.

The color analysis revealed that the optimized biscuit was darker and exhibited stronger redness and yellowness compared to the control (Figure 2). Our study clearly indicated that the inclusion of legume flours has improved the visual characteristics of the GF biscuits, potentially enhancing their consumer appeal. These results were consistent with those found by Jayasena et al. [52], who noticed a constant increase in b* both for dough and biscuits, with adding lupine flour in the formulation.

In terms of physical properties, the inclusion of legume flours has led to a decrease in the spread ratio of biscuit, reflecting changes in diameter and thickness, in baking losses, and in water activity aw, when compared to controls. Those results confirm the findings of Rababah et al. [54] and Schmelter et al. [15]. These changes reflect the structural reinforcement provided by legume proteins and fibers. Indeed, the water-binding capacity of the proteins inhibits evaporation [53]. The aw values of the GF biscuits were significantly below levels causing microbial spoilage and also below the critical point where a biscuit starts to lose its crispness during storage [55].

Moreover, the inclusion of the legume flours led to notable changes in the textural characteristics of the biscuits. Both hardness and fracturability exhibited an increase in the optimum GF biscuit (Table 5), probably due to high protein and dietary fiber contents influencing the textural properties of the biscuits. Accordingly, Schmelter et al. [15] noticed an increase in cookie hardness when the proportion of faba bean was increased from 50% to 100%. Likewise, De la Rosa-Millán et al. [56] demonstrated that a mere 10% inclusion of navy bean flour was adequate to notably elevate the hardness of GF corn flour cookies. Similarly, Yadav et al. [44] documented that the fracture strength of biscuits increased significantly (*p* < 0.05) with the addition of plantain and chickpea flours.

Altogether, our findings clearly demonstrated that the incorporation of legume flour has significantly improved the nutritional quality of the GF biscuit compared to the control, leading to higher protein, fiber, ash, and antioxidant contents. While some physical properties, such as spread ratio, were altered, these changes, along with the increased hardness, are likely attributable to the techno-functional properties of legume proteins and fibers, potentially contributing to a more texturally robust and nutritionally superior GF biscuit.

### 3.4. Sensory Properties

**Figure 3:**
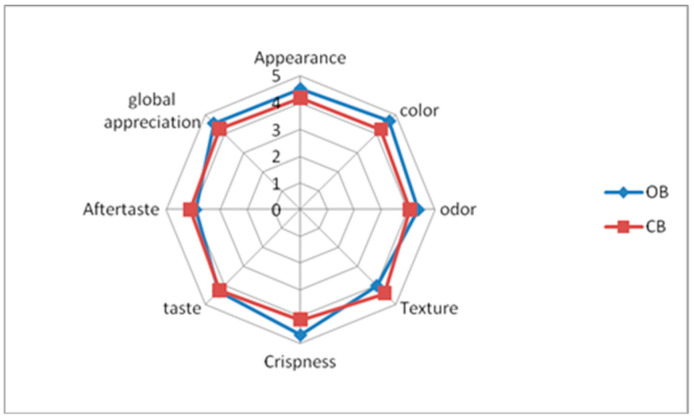
Sensory properties of the formulated GF biscuit: OB: optimum biscuit, CB: control biscuit.

The sensory analysis revealed that the optimized biscuit scored higher in appearance, color, and odor compared to the control (Figure 3). The color score corroborated instrumental color analysis, indicating the optimal biscuit appeared darker and exhibited higher values in both the yellowness and redness indexes (Table 5). However, the texture scores were slightly lower due to increased hardness, related to legume flour enrichment, confirming the instrumental determination (Table 5). Yadav et al. [44] also observed that incorporating plantain and chickpea flours has led to a firmer texture. Moreover, the inclusion of legume flour improved the crispiness score of the biscuit. Interestingly, the taste and overall acceptability scores were comparable between the optimized and control biscuits (*p* > 0.05), indicating consumer satisfaction with the enriched formulation (Figure 3). Our results are in contrast with Jayasena et al. [52], indicating a significant decrease in mean taste and flavor scores in biscuits with 30% of lupine flour substitution. A lower score of aftertaste was observed in the optimum GF biscuit compared to the controls; this could be attributed to the distinctive beany flavor associated with legume flours. In conclusion, the sensory analysis revealed that despite slight textural drawbacks, the inclusion of legume flours in the formulation of the GF biscuit had improved the sensory attributes and overall consumer acceptability. Similarly, Benkadri et al. [8] found the highest score in terms of sensory acceptability for a GF biscuit formulated from composite rice–chickpea flour.

## 4. Conclusions

This study successfully demonstrated an innovative and novel approach to improving GF biscuits by strategically utilizing local legume flour (faba bean, lentil, and chickpea) using a mixture design. This approach allowed the optimization of each parameter to obtain a GF product with predicted values for biscuit hardness, color parameter (L*, a*, and b*), water activity aw, spread ratio, and baking loss that closely matched those of a GF sample based on rice flour and maize. The inclusion of the legume flours, especially the chickpea flour, in the biscuits significantly increased their hardness and reduced aw and spread ratio, with the chickpea flour having the most substantial impact. The optimal GF formulation consists of 24.50% of faba bean flour, 19.92% of lentil flour, 6.89% of chickpea flour, 24.67% of rice flour, and 24% of maize, achieving a desirability score of 73.01%. Our findings demonstrated significant improvements in the nutritional, functional, physical quality, and antioxidant potential compared to traditional rice- and maize-based GF biscuits. Moreover, the inclusion of the legume flour blends improved global appreciation and sensory attributes of the optimized GF biscuit when compared to the control. Altogether, these improvements offer substantial benefits for consumers with celiac disease by providing more nutrient-dense, potentially healthier, and palatable alternatives to conventional GF products. In conclusion, our findings demonstrate the potential of legume flours (faba bean, lentil, and chickpea) as nutritious, appealing, sustainable, accessible, and affordable alternative ingredients for formulations of GF biscuits meeting functional and sensory standards. Further studies should consider the impact of storage on the quality of optimized legume-enriched GF biscuits to further inform their commercial viability. Future research should also deepen the nutritional and health impacts of these legume-enriched GF biscuits by examining their glycemic response, nutrient digestibility, and bioavailability.

## Figures and Tables

**Table 1:** Physicochemical characteristics of flour samples.

Flours		Faba Bean “Bachar”	Lentil “Kef”	Chickpea “Beja 1”	Rice	Maize
Moisture (g/100 g DM)		11.85 ± 0.03 ^b^	12.13 ± 0.46 ^bc^	10.40 ± 0.01 ^a^	13.36 ± 0.01 ^c^	12.18 ± 0.54 ^bc^
Carbohydrates (g/100 g DM)		55.72 ± 0.51 ^a^	57.32 ± 0.31 ^ab^	59.22 ± 0.57 ^b^	75.25 ± 0.63 ^d^	71.90 ± 0.25 ^c^
Ash (g/100 g DM)		2.37 ± 0.005 ^b^	2.43 ± 0.02 ^b^	2.64 ± 0.001 ^b^	0.71 ± 0.03 ^a^	0.95 ± 0.0004 ^a^
Protein (g/100 g DM)		28.71 ± 0.16 ^c^	26.35 ± 0.37 ^c^	22.67 ± 0.64 ^b^	10.26 ± 0.62 ^a^	10.42 ± 0.20 ^a^
Fat (g/100 g DM)		1.88 ± 0.01 ^c^	1.6 ± 0.08 ^b^	5.06 ± 0.05 ^e^	0.40 ± 0.03 ^a^	4.15 ± 0.01 ^d^
Total Polyphenols (mg GAE/g DM)		0.43 ± 0.02 ^b^	0.29 ± 0.008 ^ab^	0.65 ± 0.01 ^c^	0.2 ± 0.01 ^a^	1.04 ± 0.06 ^d^
CIE Lab Parameters	L*	92.93 ± 0.17 ^b^	85.15 ± 0.35 ^a^	93.81 ± 0.25 ^b^	93.57 ± 0.17 ^b^	85 ± 0.42 ^a^
	a*	−1 ± 0.05 ^b^	−1.21 ± 0.11 ^b^	−2.57 ± 0.02 ^a^	−0.175 ± 0.005 ^c^	−2.45 ± 0.11 ^a^
	b*	7.4 ± 0.19 ^b^	11.72 ± 0.23 ^c^	18.49 ± 0.4 ^d^	3.86 ± 0.002 ^a^	39.39 ± 0.005 ^e^
Mineral Composition	Fe (ppm)	53.97 ± 2.45 ^b^	152.45 ± 2.85 ^d^	119.75 ± 0.75 ^c^	26.98 ± 0.45 ^a^	24 ± 0.3 ^a^
	Zn (ppm)	48.22 ± 1.98 ^d^	39.18 ± 0.25 ^c^	51.28 ± 0.44 ^d^	18 ± 0.65 ^a^	25 ± 0.02 ^b^
	K (%)	1.10 ± 0.002 ^c^	0.87 ± 0.004 ^b^	1.072 ± 0.039 ^c^	0.15 ± 0.001 ^a^	0.23 ± 0.002 ^a^

Means with different superscript letters in the same row are significantly different according to Tukey’s test (*p*< 0.05). DM = dry matter.

**Table 2:** Experimental results of the analysis of physical and textural characteristics of GF biscuits.

Exp	X1 (%)	X2 (%)	X3 (%)	X4 (%)	X5 (%)	Biscuit Hardness (N)	aw	Spread Ratio	Backing Loss (%)	L*	a*	b*
1	17.50	12.50	12.50	17.25	40.25	66.00	0.34	4.26	19.94	85.71	0.50	32.86
2	25.00	20.00	6.50	24.00	24.50	55.50	0.24	3.96	18.60	73.38	2.53	35.06
3	10.00	5.00	18.50	10.50	56.00	54.00	0.20	3.09	17.35	75.54	1.11	33,62
4	10.00	20.00	5.00	10.50	54.50	44.50	0.19	3.96	18.19	76.16	0.33	35.02
5	10.00	18.50	5.00	10.50	56.00	42.50	0.19	3.89	12.60	76.66	0.75	36.21
6	25.00	5.00	5.00	10.50	54.50	54.50	0.27	3.85	16.92	73.87	2.16	35.96
7	25.00	20.00	5.00	24.00	26.00	71.50	0.28	3.86	15.81	75.26	1.85	36.88
8	25.00	5.00	20.00	10.50	39.50	49.00	0.24	3.50	9.69	76.00	1.39	35.36
9	25.00	20.00	5.00	10.50	39.50	57.00	0.24	3.86	13.44	76.35	0.86	35.45
10	10.00	2000	20.00	10.50	39.50	65.50	0.21	3.65	13.88	73.35	2.38	36.45
11	25.00	6.50	20.00	24.00	24.50	70.50	0.25	3.25	11.11	72.99	2.74	37.22
12	10.00	5.00	5.00	24.00	56.00	82.50	0.19	3.90	11.92	74.95	1.71	36.98
13	17.50	12.50	12.50	17.25	40.25	57.00	0.19	3.56	12.76	74.93	1.93	37.32
14	10.00	20.00	20.00	24.00	26.00	65.00	0.23	3.25	11.02	70.20	4.00	37.79
15	10.00	5.00	20.00	24.00	41.00	57.50	0.35	4.02	14.60	74.96	1.68	34.14
16	11.50	20.00	20.00	24.00	24.50	58.50	0.26	4.25	15.97	76.18	1.11	34.98
17	25.00	20.00	20.00	10.50	24.50	57.00	0.23	4.05	16.32	74.82	1.79	34.94
18	17.50	12.50	12.50	17.25	40.25	70.50	0.21	3.41	15.46	74.88	2.030	36.75
19	10.00	20.00	5.00	24.00	41.00	39.50	0.21	3.75	14.78	72.54	2.73	36.34
20	25.00	5.00	5.00	24.00	41.00	57.00	0.26	3.40	13.83	74.45	1.72	35.87
21	25.00	5.00	20.00	24.00	26.00	55.50	0.37	3.96	13.78	75.05	1.57	34.83
22	23.50	5.00	5.00	10.50	56.00	54.50	0.39	3.95	11.27	74.99	1.46	34.96
23	10.00	5.00	20.00	10.50	54.50	58.00	0.39	3.68	11.10	74.81	1.69	35.29

The experiments from 1 to 23 represent the distinct biscuit formulation combinations generated by the mixture design. X1: faba bean; X2: lentil; X3: chickpea; X4: rice; X5: maize. aw: water activity.

**Table 3:** Proposed model validation parameters.

	Sum of Squares	F	Significance	R^2^_Adj_ (%)	R^2^ (%)
Biscuit hardness (N)	1156.90	5.17	**	43.12	53.46
Spread ratio	4.35	475.60	***	99.76	99.97
Baking loss (%)	157.33	6.63	*	80.37	94.65
Water activity (aw)	0.09	35.64	***	95.94	98.71
L*	162.99	262.79	***	99.51	99.89
a*	14.69	90.88	***	98.66	99.76
b*	32.99	82.05	***	98.22	99.43

*: *p* < 0.05; **: *p* < 0.01; ***: *p* < 0.001.

**Table 4:** Desirability results and optimal formulation parameters.

Responses	Target	Values	Desirability (%)	Factors	Real Values (%)
Biscuit Hardness (N)	55.00	61.04	63.35	Faba bean flour	24.50
Spread Ratio	4.45	4.43	97.28	Lentil flour	19.92
Backing Loss (%)	15.00	15.68	86,16	Chickpea flour	6.89
aw	0.28	0. 27	83. 93	Rice flour	24.67
L*	80.00	75.17	50.72	Maize flour	24
a*	3.00	1.43	37.22		
b*	35.00	35.09	96.55		
Total Desirability			**73.01**		

**Table 5:** Physicochemical analysis of the GF optimum biscuit and the control.

	Optimum	Control
Protein (g/100 g DM)	11.90 ± 0.09 ^b^	6.46 ± 0.12 ^a^
Fat (g/100 g DM)	16.50 ± 0.11 ^a^	16.37 ± 0.08 ^a^
Ash (g/100 g DM)	1.25 ± 0.02 ^b^	0.58 ± 0.06 ^a^
Moisture (%)	7.69 ± 0.06 ^b^	7.35 ± 0.01 ^a^
Insoluble Dietary Fiber (g/100 g DM)	16.85 ± 1.06 ^b^	12.80 ± 0.63 ^a^
Total Dietary Fiber (g/100 g DM)	21.13 ± 0.38 ^b^	15.73 ± 0.64 ^a^
Carbohydrates (g/100 g DM)	41.54 ± 0.59 ^a^	53.45 ± 0.89 ^b^
Diameter (mm)	42.16 ± 0.26 ^a^	43.75 ± 0.14 ^b^
Thickness (mm)	8.86 ± 0.08 ^a^	8.90 ± 0,10 ^a^
Spread Ratio	4.75 ± 0.01 ^a^	4.91 ± 0.07 ^a^
Baking Loss (%)	10.63 ± 0.03 ^a^	12.93 ± 0.26 ^b^
aw	0.17± 0.0005 ^a^	0.23± 0.001 ^b^
L*	71.07 ± 1.53 ^a^	79.74 ± 0.62 ^b^
a*	1.73 ± 0.04 ^b^	−0.72 ± 0.04 ^a^
b*	36.38 ± 0.04 ^b^	31.33 ± 0.09 ^a^
Hardness (N)	34.66 ± 3.68 ^b^	22.00 ± 1.41 ^a^
Fracturability (mm)	0.98 ± 0.098 ^b^	0.74 ± 0.07 ^a^
Dough Hardness (N)	6.95 ± 0.03 ^b^	5.79 ± 0.07 ^a^
Total Polyphenol (mg GAE/g DM)	0.34 ± 0.01 ^b^	0.18 ± 0.006 ^a^
DPPH (%)	72.72 ± 0.32 ^b^	31.49 ± 4.54 ^a^

Means with different superscript letters in the same row are significantly different according to Tukey’s test (*p* < 0.05). DM = dry matter.

## Data Availability

The original contributions presented in the study are included in the article; further inquiries can be directed to the corresponding author.

## Abbreviations

The following abbreviations are used in this manuscript:

GF	Gluten-free
aw	Water Activity
TPP	Total Polyphenol
GA	Gallic Acid
GAE	Gallic Acid Equivalent
DM	Dry Matter
